# Development of Serotonergic Fibers in the Post-Natal Mouse Brain

**DOI:** 10.3389/fncel.2017.00202

**Published:** 2017-07-14

**Authors:** Giacomo Maddaloni, Alice Bertero, Marta Pratelli, Noemi Barsotti, Annemarie Boonstra, Andrea Giorgi, Sara Migliarini, Massimo Pasqualetti

**Affiliations:** ^1^Unit of Cell and Developmental Biology, Department of Biology, University of Pisa Pisa, Italy; ^2^Center for Neuroscience and Cognitive Systems, Istituto Italiano di Technologia, University of Trento Rovereto, Italy

**Keywords:** serotonin, axonal morphology, post-natal development, innervation heterogeneity, 3D-reconstruction, mouse models

## Abstract

Serotonin (5-HT)-synthetizing neurons, which are confined in the *raphe* nuclei of the rhombencephalon, provide a pervasive innervation of the central nervous system (CNS) and are involved in the modulation of a plethora of functions in both developing and adult brain. Classical studies have described the post-natal development of serotonergic axons as a linear process of terminal field innervation. However, technical limitations have hampered a fine morphological characterization. With the advent of genetic mouse models, the possibility to label specific neuronal populations allowed the rigorous measurement of their axonal morphological features as well as their developmental dynamics. Here, we used the *Tph2*^GFP^ knock-in mouse line, in which GFP expression allows punctual identification of serotonergic neurons and axons, for confocal microscope imaging and we performed 3-dimensional reconstruction in order to morphologically characterize the development of serotonergic fibers in specified brain targets from birth to adulthood. Our analysis highlighted region-specific developmental patterns of serotonergic fiber density ranging from a linear and progressive colonization of the target (Caudate/Putamen, Basolateral Amygdala, Geniculate Nucleus and Substantia Nigra) to a transient increase in fiber density (medial Prefrontal Cortex, Globus Pallidus, Somatosensory Cortex and Hippocampus) occurring with a region-specific timing. Despite a common pattern of early post-natal morphological maturation in which a progressive rearrangement from a dot-shaped to a regular and smooth fiber morphology was observed, starting from post-natal day 28 serotonergic fibers acquire the region specific morphological features present in the adult. In conclusion, we provided novel, target-specific insights on the morphology and temporal dynamics of the developing serotonergic fibers.

## Introduction

The whole central nervous system (CNS) of vertebrates is reached and profusely innervated by serotonin (5-hydroxytryptamine; 5-HT) releasing fibers. Such a diffuse distribution of serotonergic axons arises entirely from a relatively small number of somata (approximately 28,000 in the mouse; Ishimura et al., 1988) that are confined in the brainstem and clustered in B1 to B9 *raphe* nuclei. Serotonergic neurons represent one of the first neuronal systems to be specified during development (Levitt and Rakic, 1982). In the mouse, as early as embryonic day 11.5 (E11.5) the newly specified neurons start to elongate their axons rostrally in the medial forebrain bundle. Starting from E13.5, serotonergic fibers are driven along the main brain trajectories, reaching their targets by the end of gestation (Lidov and Molliver, 1982; Gaspar et al., 2003). The subsequent post-natal terminal field development has been described as a gradual colonization of the target with a region-specific timing (Lidov and Molliver, 1982). In the adult, 5-HT fibers display at least two different morphologies, originating from Dorsal (D-fibers) or Median (M-fibers) *raphe* nuclei. D-fibers appear thin with fusiform homogeneous varicosities and are more abundant than M-fibers, which show larger and oval varicosities along thin axons (Kosofsky and Molliver, 1987; Mamounas and Molliver, 1988; Wilson et al., 1989; Törk, 1990; Bang et al., 2012).

Classically, 5-HT immunolabeling has been used to study serotonergic fiber morphology (Lidov and Molliver, 1982; Azmitia and Gannon, 1983; D’Amato et al., 1987; Törk, 1990; Nyakas et al., 1994). However, as 5-HT is released and rapidly metabolized, reliable 5-HT immunostaining is not easily achievable thus requiring L-tryptophan and MAO-A inhibitors administration before tissue harvesting (Lidov and Molliver, 1982; Azmitia and Gannon, 1983; D’Amato et al., 1987; Nyakas et al., 1994). Alternatively, 5-HT transporter (SERT) immunohistochemistry has been used (Belmer et al., 2017). However, in the adult brain SERT immunostaining partially overlaps with serotonergic fibers since SERT is not present on their whole extent (Brown and Molliver, 2000; Amilhon et al., 2010; Descarries et al., 2010). Furthermore, SERT is transiently expressed in non-serotonergic neurons in the developing brain (Lebrand et al., 1998; Narboux-Nême et al., 2008).

Here we used the *Tph2*^*GFP*^ knock-in mouse line in which *Tph2*, the rate-limiting enzyme of 5-HT synthesis, has been replaced by the enhanced GFP reporter that accumulates and freely diffuses within the cytoplasm of all 5-HT synthetizing neurons, without affecting brain 5-HT levels or serotonergic system development and organization (Migliarini et al., 2013). This ensures an easy and high fidelity visualization of the fine-grain anatomy of the whole 5-HT system, which already allowed us to study different aspects of 5-HT fiber dynamics, both *in vitro* and *in vivo*, as well as during mouse development and in adulthood (Migliarini et al., 2013; Pelosi et al., 2015; Pacini et al., 2017; Pratelli et al., 2017). We combined GFP immunofluorescence with confocal microscope imaging and 3D-reconstruction to quantitatively characterize the post-natal development of serotonergic fibers in selected brain regions. Our approach allowed the characterization of previously unreported changes in regional density and morphology of 5-HT axons that occur in a time- and region-specific manner.

## Material and Methods

### Animals and Immunohistochemistry

Animals were maintained on artificial 12/12 h light/dark cycle at constant temperature of 22 ± 1°C and housed in standard Plexiglas cages with food and water *ad libitum*. All experimental protocols were conducted in accordance with the Ethics Committee of the University of Pisa and approved by the Veterinary Department of the Italian Ministry of Health. In the present study, three *Tph2*^*GFP*^ heterozygous males per stage were used. The analyzed time-points were post-natal day 0 (PND 0), PND 7, PND 14, PND 28 and 20 weeks old (adult).

Deeply anesthetized (avertin, i.p. 250 mg/kg) animals were transcardially perfused with phosphate buffered saline, followed by 4% paraformaldehyde (PFA). Brains were dissected and post-fixed over night at 4°C in 4% PFA. 50 μm (PND 14, PND 28, Adult) or 100 μm (PND 0, PND 7) coronal sections were obtained with a vibratome (Leica Microsystems). Immunohistochemical procedures were performed on free floating sections as described in Migliarini et al. (2013), using chicken anti-GFP (1:1000, AbCam) primary antibody, followed by Alexa Fluor 488 goat anti chicken IgG (1:500, Life Technologies) secondary antibody.

### Image Acquisition, 3D-Reconstruction and Statistical Analysis

Though serotonergic axons provide widespread innervation to virtually every region of the CNS, in the present study we focused our analysis on selected regions of the fore- and mid-brain. Specifically, we analyzed eight brain districts that are involved in distinct functions including control of locomotion, such as the Caudate/Putamen (CPu), the Globus Pallidus (GP) and the Substantia Nigra (SN); sensory perception, such as the Primary Somatosensory Cortex (Barrel Field, S1BF, layer IV) and the Dorsal Lateral Geniculate Nucleus (DLG); cognitive and emotional processing, such as the medial Prefrontal Cortex (mPFC, layer V), the Basolateral Amygdala (BLA) and the dorsal Hippocampus (Hp, *Lacunosum moleculare* layer of the dorsal CA1). For each region and stage (*n* = 3 animals), two high power confocal images on adjacent sections were acquired on a Nikon A1 confocal system, using a 60× plan-apochromat objective. Z series of 69 stacks were acquired at 1024 × 1024 pixel resolution (pixel size: 0.21 μm), with a z-step of 0.15 μm. For each acquisition, 3D-reconstruction analysis was performed on three blocks of 300 pixel × 300 pixel × 69 stacks (*xyz* = 63 × 63 × 10 μm, 39.69 × 10^3^ μm^3^) using the semi-automatic Filament tool of IMARIS software (Bitplane). Each reconstructed block was then manually corrected for false segments by multiple operators, which were blinded on the brain region and the stage under analysis. Total volume, total length, filament mean diameter and edge diameter (ED) values were extracted from IMARIS output and plotted with GraphPad Prism 6.0 software. A total of six blocks per structure was averaged to generate a group mean and SEM (*n* = 3 animals per region). Intra-stage comparisons were statistically validated by one-way ANOVA with Tukey’s correction for multiple comparisons. The resulting *p-values* were represented as heatmaps flanking their corresponding graphs. Inter-stage log_2_ fold changes for PND 7, PND 14 and PND 28 were calculated for each region as compared to their previous developmental stage, with statistical significance reported in the graphs assessed with two tailed Student’s *t*-test. Not significant: *p* > 0.05; *p < 0.05; **p < 0.01; ***p < 0.001; ****p < 0.0001.

## Results

**Figure 1:**
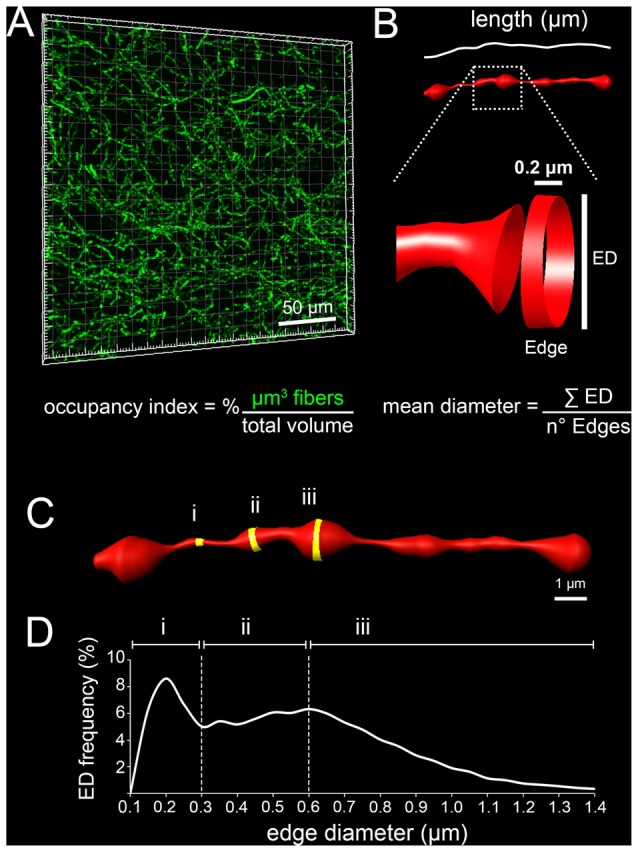
Schematic representation of the parameters used to characterize serotonergic fibers. **(A)** Occupancy index defined as the percentage of μm^3^ occupied by serotonergic fibers in the analyzed block.** (B)** Total fiber length expressed as μm of fibers passing within the analyzed block. A portion of the reconstructed fiber is enlarged to provide a schematic representation of an edge that is the smallest unit, 0.2 μm in length, generated by IMARIS to build 3D fibers. The diameter of edges (ED) was used to calculate the mean diameter of fibers. **(C)** Representative 3D-reconstruction in which thin (i), intermediate (ii) and thick (iii) edges are highlighted in yellow. **(D)** Example of a typical ED frequency curve in which the diameter intervals of thin (i), intermediate (ii) and thick (iii) edges are reported.

The possibility to use a knock-in replacement strategy in the mouse allows the labeling of specific neuronal populations together with their axonal projections and the analysis of their dynamic processes during development (Pasqualetti et al., 2002). Here we have successfully used the *Tph2*^*GFP*^ knock-in mouse line (Migliarini et al., 2013) to label and study the region-specific organization of serotonergic fibers in the developing post-natal mouse brain. We measured discrete parameters including the occupancy index (% of volume occupied by serotonergic fibers; Figure 1A), the total length of fibers passing in the analyzed block of brain tissue and the mean diameter of 5-HT axons (Figure 1B). Moreover, as serotonergic fibers are characterized by alternating thin and thick segments, the diameter of each edge, 0.2 μm long units generated by IMARIS to build 3D fibers (Figures 1B–C), was used to draw an ED distribution curve (Figure 1D).

**Figure 2:**
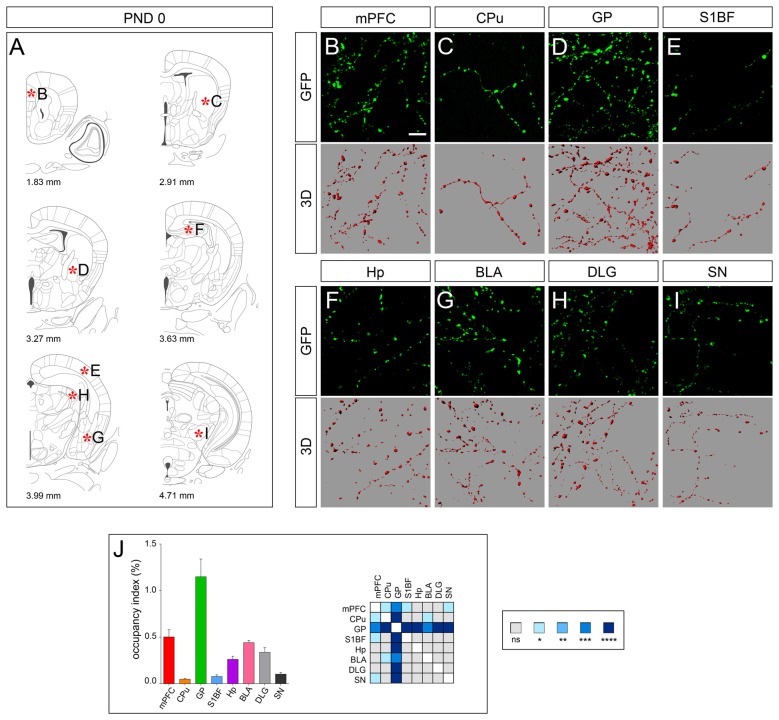
3D-reconstruction and analysis of serotonergic fibers in post-natal day 0 (PND 0) brain. **(A)** Coronal tables adapted from the atlas of developing mouse brain showing the precise anatomical localization of the region analyzed (Paxinos et al., 2007), as indicated by asterisks corresponding to high magnification images in **(B–I)**. **(B–I)** Representative high power confocal images of GFP-immunostained serotonergic fibers of a single analyzed block, and their 3D-reconstructions. **(J)** Histogram comparing the occupancy index across the regions, and heatmap showing color-coded *p*-values of the differences in occupancy index among the analyzed regions. Data are expressed as mean ± SEM. Statistical significance was calculated with one way ANOVA with multiple comparisons and Tukey’s *p-values* correction. ns: *p* > 0.05; *p < 0.05; **p < 0.01; ***p < 0.001; ****p < 0.0001. Scale bar: 10 μm. mPFC, medial Prefrontal Cortex; GP, Globus Pallidus; CPu, Caudate/Putamen; S1BF, Primary Somatosensory Cortex, Barrel Field; BLA, Basolateral Amygdala; Hp, *Lacunosum moleculare* layer of the dorsal CA1 of hippocampus; DLG, Dorsal Lateral Geniculate Nucleus; SN, Substantia Nigra.

Analysis at PND 0, when serotonergic fibers have reached their targets in the rostral brain, revealed a marked difference in the density of GFP-immunoreactive fibers among the analyzed areas that were further highlighted by 3D-reconstruction (Figures 2A–I). In particular CPu, S1BF and SN showed the lowest occupancy index (e.g., less than 0.1%) and GP the highest (i.e., 1.15 ± 0.19%; Figure 2J). On the other hand, we observed a rather homogeneous morphology of 5-HT fibers, which were characterized by a dot shape (Figures 2B–I). This was particularly evident in mPFC, GP, Hp, BLA, DLG and SN, in which the segments connecting the dot-shaped structures were barely detectable (Figures 2B,D,F–H). As a consequence, such a discontinuous profile did not allow to perform further fiber measurements.

**Figure 3:**
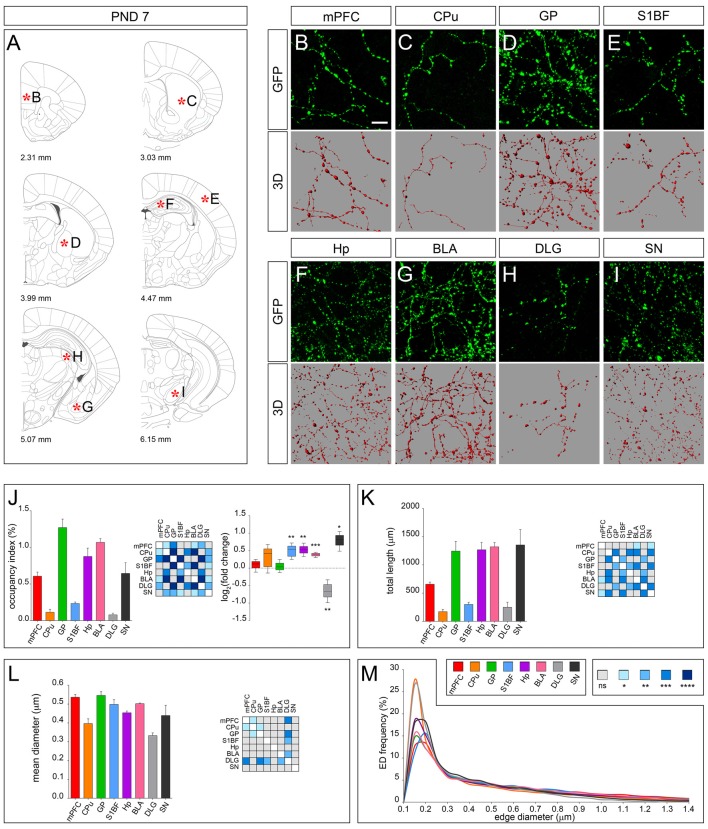
3D-reconstruction and analysis of serotonergic fibers in PND 7 brain. **(A)** Coronal tables adapted from the atlas of developing mouse brain showing the precise anatomical localization of the region analyzed (Paxinos et al., 2007), as indicated by asterisks corresponding to high magnification images in **(B–I)**. **(B–I)** Representative high power confocal images of GFP-immunostained serotonergic fibers of a single analyzed block, and their 3D-reconstructions. **(J)** Histogram comparing the occupancy index across the regions, and heatmap showing color-coded *p*-values of the differences in occupancy index among the analyzed regions. Box plots show the log_2_ fold changes increase/decrease in the occupancy index at PND 7, as compared to PND 0. **(K)** Histogram showing the total length of 5-hydroxytryptamine (5-HT)-fibers in the analyzed areas, and heatmap representing color-coded *p-values* relative to the differences in fiber length among the regions. **(L)** Histogram showing the mean diameter of serotonergic fibers, and heatmap representing color-coded *p-value* relative to the differences in fiber diameter. **(M)** ED distribution graph, composed by curves depicting the frequency of (EDs; from 0.1 μm to 1.4 μm, 0.05 μm step; *x*-axis) in each region. Data are expressed as mean ± SEM. Statistical significance was calculated with one way ANOVA, with multiple comparisons and Tukey’s *p-values* correction. Two tailed Student’s *t* test for unpaired data statistical significance was reported in box-whisker graphs. ns: *p* > 0.05; *p < 0.05; **p < 0.01; ***p < 0.001; ****p < 0.0001. Scale bar: 10 μm. mPFC, medial Prefrontal Cortex; GP, Globus Pallidus; CPu, Caudate/Putamen; S1BF, Primary Somatosensory Cortex, Barrel Field; BLA, Basolateral Amygdala; Hp, *Lacunosum moleculare* layer of the dorsal CA1 of hippocampus; DLG, Dorsal Lateral Geniculate Nucleus; SN, Substantia Nigra.

At PND 7, connecting segments became detectable in all the analyzed brain districts while the dot shape was maintained, and an overall increase of fiber density was observed in line with the progression of terminal field development (Figures 3A–I). This trend was confirmed by 3D-reconstruction showing an increase in the occupancy index, which was particularly evident for S1BF, Hp, BLA and SN (Figure 3J), with the exception of DLG in which a significant decrease was observed (log_2_ fold change −0.66; Figure 3J). The possibility to detect the connecting segments allowed to measure the total fiber length (Figure 3K), and the mean diameter (Figure 3L). Notably, a similar distribution of all ED curves was observed (Figure 3M).

**Figure 4:**
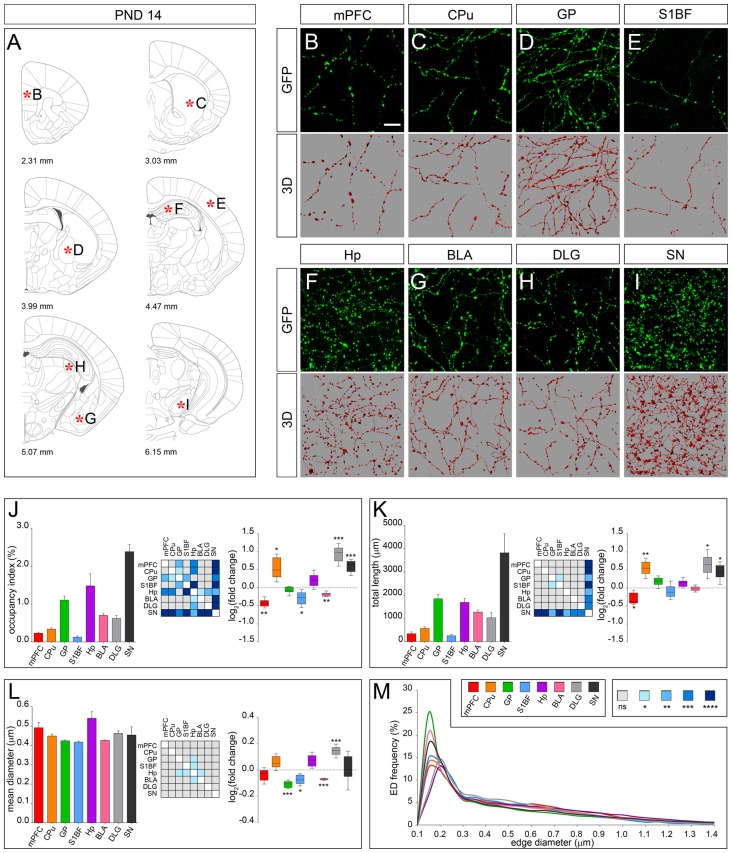
3D-reconstruction and analysis of serotonergic fibers in PND 14 brain. **(A)** Coronal tables adapted from the atlas of developing mouse brain showing the precise anatomical localization of the region analyzed (Paxinos et al., 2007), as indicated by asterisks corresponding to high magnification images in **(B–I)**. **(B–I)** Representative high power confocal images of GFP-immunostained serotonergic fibers of a single analyzed block, and their 3D-reconstructions. **(J)** Histogram comparing the occupancy index across the regions, and heatmap showing color-coded *p*-values of the differences in occupancy index among the analyzed regions. Box plots show the log_2_ fold changes increase/decrease in the occupancy index at PND 14, as compared to PND 7. **(K)** Histogram showing the total length of 5-HT-fibers in the analyzed areas, and heatmap representing color-coded *p-values* relative to the differences in fiber length among the regions. Box plots show log_2_ fold change increase/decrease in fiber length in each region at PND 14, as compared to PND 7. **(L)** Histogram showing the mean diameter of serotonergic fibers, and heatmap representing color-coded *p-value* relative to the differences in fiber diameter. Box plots show the log_2_ fold changes increase/decrease in the mean diameter calculated at PND 14, as compared to PND 7. **(M)** ED distribution graph, composed by curves depicting the frequency of edge diameters (from 0.1 μm to 1.4 μm, 0.05 μm step; *x*-axis) in each region. Data are expressed as mean ± SEM. Statistical significance was calculated with one way ANOVA, with multiple comparisons and Tukey’s *p-values* correction. Two tailed Student’s *t* test for unpaired data statistical significance was reported in box-whisker graphs. ns: *p* > 0.05; *p < 0.05; **p < 0.01; ***p < 0.001; ****p < 0.0001. Scale bar: 10 μm. mPFC, medial Prefrontal Cortex; GP, Globus Pallidus; CPu, Caudate/Putamen; S1BF, Primary Somatosensory Cortex, Barrel Field; BLA, Basolateral Amygdala; Hp, *Lacunosum moleculare* layer of the dorsal CA1 of hippocampus; DLG, Dorsal Lateral Geniculate Nucleus; SN, Substantia Nigra.

The morphology of serotonergic fibers appeared more uniform at PND 14 (Figures 4A–I). Interestingly, 3D-reconstruction revealed divergent developmental patterns among the analyzed areas. Fibers innervating CPu, DLG and SN exhibited a significant increase in occupancy index (log_2_ fold change +0.53, +0.93 and +0.59, respectively) and total length (log_2_ fold change +0.54, +0.65 and +0.45, respectively) as compared to PND 7. A similar trend was observed in Hp, although not statistically significant. By contrast, fibers present in mPFC, S1BF and BLA showed a reduced occupancy index (log_2_ fold change −0.42, −0.29 and −0.18, respectively; Figure 4J), which can be ascribed to changes in total length (i.e., mPFC, log_2_ fold change −0.30) and mean diameter (i.e., S1BF and BLA, log_2_ fold change −0.08 and −0.07, respectively; Figures 4K–M). Finally, no significant changes were detected in serotonergic fibers innervating GP (Figures 4J–M).

**Figure 5:**
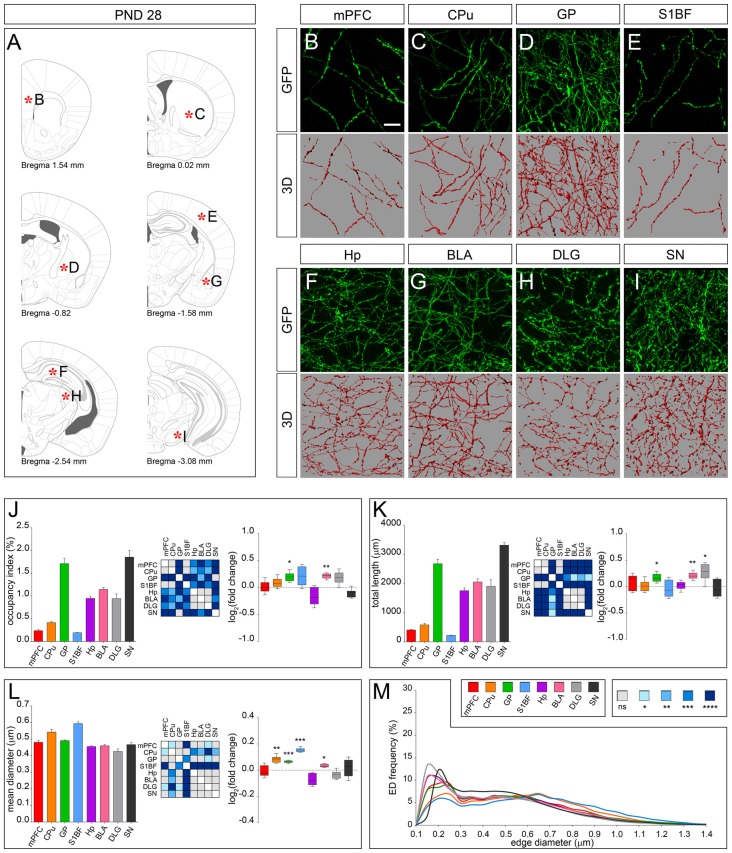
3D-reconstruction and analysis of serotonergic fibers in PND 28 brain. **(A)** Coronal tables adapted from adult mouse brain atlas showing the precise anatomical localization of the region analyzed (Franklin and Paxinos, 2008), as indicated by asterisks corresponding to high magnification images in **(B–I)**. **(B–I)** Representative high power confocal images of GFP-immunostained serotonergic fibers of a single analyzed block, and their 3D-reconstructions. **(J)** Histogram comparing the occupancy index across the regions, and heatmap showing color-coded *p*-values of the differences in occupancy index among the analyzed regions. Box plots show the log_2_ fold changes increase/decrease in the occupancy index at PND 28, as compared to PND 14. **(K)** Histogram showing the total length of 5-HT-fibers in the analyzed areas, and heatmap representing color-coded *p-values* relative to the differences in fiber length among the regions. Box plots show log_2_ fold change increase/decrease in fiber length in each region at PND 28, as compared to PND 14. **(L)** Histogram showing the mean diameter of serotonergic fibers, and heatmap representing color-coded *p-value* relative to the differences in fiber diameter. Box plots show the log_2_ fold changes increase/decrease in the mean diameter calculated at PND 28, as compared to PND 14. **(M)** ED distribution graph, composed by curves depicting the frequency of EDs (from 0.1 μm to 1.4 μm, 0.05 μm step; *x*-axis) in each region. Data are expressed as mean ± SEM. Statistical significance was calculated with one way ANOVA, with multiple comparisons and Tukey’s *p-values* correction. Two tailed Student’s *t* test for unpaired data statistical significance was reported in box-whisker graphs. ns: *p* > 0.05; *p < 0.05; **p < 0.01; ***p < 0.001; ****p < 0.0001. Scale bar: 10 μm. mPFC, medial Prefrontal Cortex; GP, Globus Pallidus; CPu, Caudate/Putamen; S1BF, Primary Somatosensory Cortex, Barrel Field; BLA, Basolateral Amygdala; Hp, *Lacunosum moleculare* layer of the dorsal CA1 of hippocampus; DLG, Dorsal Lateral Geniculate Nucleus; SN, Substantia Nigra.

The dot-shaped feature observed at earlier stages was lost at PND 28 when 5-HT fibers display a uniform and smooth appearance (Figures 5A–I). As compared to PND 14, fibers in GP and BLA showed a marked increase in the occupancy index (Figure 5J), total length (Figure 5K) and an increase in their mean diameter (Figures 5L,M). A similar rearrangement was also apparent in DLG, CPu and S1BF due to changes in total length (i.e., DLG) or in mean diameter (i.e., CPu and S1BF). mPFC, Hp and SN displayed subtle fluctuation of the analyzed parameters but showed no significant change in the occupancy index as compared to PND 14 (Figures 5J–L). On the whole, the regional specificity of the morphological development observed between PND 14 and PND 28 accounted for the identification of two main fiber classes with distinctive features, as highlighted by ED curves (Figure 5M). In fact, higher frequency of edges in the diameter range of 0.15–0.3 μm was found in serotonergic fibers present in mPFC, GP, BLA, HP, DLG and SN as compared to CPu and S1BF. Conversely, CPu and S1BF displayed higher frequency of edges, whose diameter was >0.6 μm.

**Figure 6:**
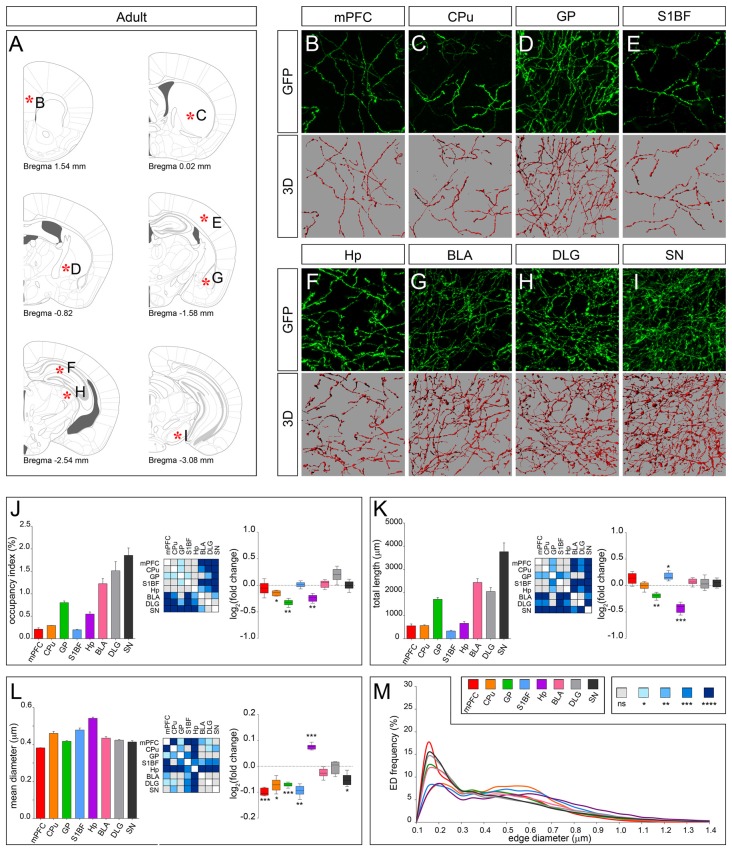
3D-reconstruction and analysis of serotonergic fibers in the adult brain. **(A)** Coronal tables adapted from the mouse brain atlas showing the precise anatomical localization of the region analyzed (Franklin and Paxinos, 2008), as indicated by asterisks corresponding to high magnification images in **(B-I)**. **(B–I)** Representative high power confocal images of GFP-immunostained serotonergic fibers of a single analyzed block, and their 3D-reconstructions. **(J)** Histogram comparing the occupancy index across the regions, and heatmap showing color-coded *p*-values of the differences in occupancy index among the analyzed regions. Box plots show the log_2_ fold changes increase/decrease in the occupancy index in adult, as compared to PND 28. **(K)** Histogram showing the total length of 5-HT-fibers in the analyzed areas, and heatmap representing color-coded *p-values* relative to the differences in fiber length among the regions. Box plots show log_2_ fold change increase/decrease in fiber length in each region in the adult, as compared to PND 28. **(L)** Histogram showing the mean diameter of serotonergic fibers, and heatmap representing color-coded *p-value* relative to the differences in fiber diameter. Box plots show the log_2_ fold changes increase/decrease in the mean diameter calculated in the adult, as compared to PND 28. **(M)** ED distribution graph, composed by curves depicting the frequency of EDs (from 0.1 μm to 1.4 μm, 0.05 μm step; *x*-axis) in each region. Data are expressed as mean ± SEM. Statistical significance was calculated with one way ANOVA, with multiple comparisons and Tukey’s *p-values* correction. Two tailed Student’s *t* test for unpaired data statistical significance was reported in box-whisker graphs. ns: *p* > 0.05; *p < 0.05; **p < 0.01; ***p < 0.001; ****p < 0.0001. Scale bar: 10 μm. mPFC, medial Prefrontal Cortex; GP, Globus Pallidus; CPu, Caudate/Putamen; S1BF, Primary Somatosensory Cortex, Barrel Field; BLA, Basolateral Amygdala; Hp, *Lacunosum moleculare* layer of the dorsal CA1 of hippocampus; DLG, Dorsal Lateral Geniculate Nucleus; SN, Substantia Nigra.

According to the classical view, serotonergic fibers in rodents complete their terminal field development within 1 month after birth (Lidov and Molliver, 1982). In line, total fiber length measured at PND 28 in mPFC, CPu, BLA, DLG and SN was maintained up to adulthood (Figures 6A–I). However, morphology of fibers in mPFC, CPu and SN was clearly remodeled, displaying a significant reduction in mean diameter in the adult as compared to PND 28 (Figure 6L). In GP and S1BF the mean diameter was reduced as well (Figure 6L). While in GP both occupancy index and total length were significantly reduced, in S1BF the occupancy index was unchanged due to an increase in total length (Figures 6J,K). A further discrepancy from the classical view was observed in Hp where serotonergic fibers remarkably decreased in total length and became thicker (Figures 6K,L). Finally, as shown by the ED curves, the regional differences in axonal morphology observed at PND 28 were exacerbated in the adult with the exception of Hp, whose ED distribution approached that of CPu and S1BF (Figure 6M). On the whole, these data demonstrated for the first time that the establishment of target-specific heterogeneity in serotonergic fibers proceeds beyond the first month after birth, thus extending the time window of terminal field development.

## Discussion

Serotonergic fiber development in the post-natal brain involves a massive and progressive increase in the amount of fibers within the target region that is commonly referred to as terminal field development (Lidov and Molliver, 1982). Measuring the total length of GFP-immunoreactive serotonergic fibers, we could identify two distinct developmental patterns showing either a progressive or a transient increase in fiber length from early post-natal stages up to adulthood, with a region-specific timing. Examples of the first scenario were observed in CPu, BLA, DLG and SN, where the total length of serotonergic fibers increased starting from PND 7 (BLA and SN) or PND 14 (CPu and DLG), peaked at PND 28 and remained unchanged up to adulthood. The second scenario was characterized by two distinct temporal trends. In cortical regions (mPFC, S1BF) serotonergic innervation peaked at PND 7, decreased up to PND 28 and, limited to the S1BF, increased again in the adult brain. In GP and Hp serotonergic innervation transiently increased up to PND 28 and then appeared pruned in the adult. Given the well-established role of 5-HT signaling in brain development (Teissier et al., 2017), and given that the density of serotonergic fibers could be directly linked to 5-HT levels, the presence of two distinct and region-specific patterns of serotonergic fiber development may account for different developmental roles of 5-HT. In particular, a transient increase of serotonergic fibers could reflect the requirement of higher 5-HT levels within time-windows that may be critical for the establishment and refinement of local neuronal circuitry. An example is provided by 5-HT fibers in the somatosensory cortex highlighting a transient increase in serotonergic innervation followed by a drop at PND 10 (Fujimiya et al., 1986; D’Amato et al., 1987). It has been postulated that this transient increase of serotonergic fibers, likely resulting in elevated 5-HT levels, could be linked to the correct establishment of thalamocortical pathway. Accordingly, bidirectional alterations of serotonergic signaling result in abnormal barrel field formation and a reduced cortical thickness (Miceli et al., 2013; Narboux-Nême et al., 2013). This suggests that a proper balance in serotonergic neurotransmission is required for the correct post-natal development of cortical regions. In this view, the transient increase in serotonergic innervation observed in Hp could be involved in controlling the intense post-natal developmental events (Angevine, 1965), such as the massive neurogenesis taking place during the first three post-natal weeks (reviewed in Reznikov, 1991). Similarly, the peak of 5-HT innervation observed in GP at PND 28 could play a role in the refinement of basal ganglia circuitry, as already demonstrated for dopamine levels during post-natal development (Sivam et al., 1991).

In the present study, we also provided a fine morphological characterization of the serotonergic fiber development in the postnatal mouse brain, showing that it proceeds in a comparable manner among all the regions analyzed. In fact, at birth fibers presented thick and dot-shaped enlargements with few and often barely detectable connections, in line with previous reports (D’Amato et al., 1987). Subsequently, thin connecting segments were detectable and fibers progressively became more smooth and uniform along their length up to PND 28. Eventually, additional intrinsic rearrangements resulted in the acquisition of the morphology observed in the adult. Our measurements display some discrepancies as compared to those obtained by Belmer et al. (2017), who also used a 3D-reconstruction approach to assess the morphology of SERT immunoreactive axons in the limbic system of adult mice. The use of SERT rather than GFP immunohistochemistry may likely account for the discrepancies observed as SERT not fully overlaps with serotonergic fibers (Brown and Molliver, 2000; Amilhon et al., 2010; Descarries et al., 2010). Thanks to the ED distribution analysis of GFP-immunoreactive serotonergic fiber, we were able to distinguish two main trends likely reflecting the presence of M and D fibers described by Kosofsky and Molliver (Kosofsky and Molliver, 1987; Törk, 1990). Accordingly, the first trend included serotonergic fibers present in mPFC, GP, BLA, DLG and SN that showed the lowest mean diameter, in line with their dorsal *raphe* origin (Muzerelle et al., 2016). However, despite the presence of fibers originating from serotonergic neurons located in the median *raphe* such as those innervating the Hp, the second included CPu and S1BF as well, which receive inputs from the dorsal *raphe* (Muzerelle et al., 2016). Nevertheless, since ED graphs depict the frequency of a given diameter describing the mean shape of fibers in each region, a specific fiber morphology (e.g., M vs. D) could not be associated to a precise curve trend. Further studies, including single fiber reconstruction approaches, are required to solve this issue.

Altogether, our data describe the general route of maturation of ascending serotonergic projections, which invade their targets with thick, dot-shaped fibers that progressively become smooth and uniform along their length, acquiring their final region specific morphology only after PND 28. Despite this common behavior in morphological rearrangements, we identified previously unreported maturation patterns that may underlie specific developmental roles for 5-HT within critical time-windows.

## Author Contributions

GM, ABe, MPr, NB, ABo, AG and SM performed experiments; analyzed data. GM, ABe, MPr and MPa interpreted data and wrote the article. MPa conceived the work.

## Conflict of Interest Statement

The authors declare that the research was conducted in the absence of any commercial or financial relationships that could be construed as a potential conflict of interest. The reviewer LC and handling Editor declared their shared affiliation, and the handling Editor states that the process nevertheless met the standards of a fair and objective review.
